# Gonorrhœa and Straight Homœopathy

**Published:** 1891-03

**Authors:** Thomas Skinner


### GONORRHŒA AND "STRAIGHT HOMŒOPATHY."

Editors of The Homœopathic Physician:—If I understand aright, Dr. T. F. Allen has the tuition of lady doctors as well as gentlemen doctors, and it would be well to know if he teaches the former as well as the latter, to use urethral and vaginal injections for the radical cure of gonorrhœa in its early stage in men or women, the disease being common to both—although, like Adam and Eve, the one generally blames the other.

It is all very well for Dr. T. F. Allen, who has to teach "the young idea how to shoot," to give them a stone when they expect bread, but when he goes into print and lays before the profession his treatment of primary gonorrhœa, *and before men of half a century of practice,* he need not feel surprised if he is severely "hauled over the coals."

In the January number of The Homœopathic Physician, Dr. Kimball has stated all that need be stated—to all of which I cordially agree—but I should like to add a parting salute to so distinguished a physician as Dr. T. F. Allen, who, by this time in his professional existence, ought to know better.

Given a man or a woman, who, by an illicit connection, has contracted a gonorrhœal discharge from the urethra, and which, be it remembered, does not appear sooner or later than from one to seven days after the impure coitus, and which must have leavened the entire system, in the interval, with the virus (the whole man or woman), are we to believe that, by a *local* injection of anything from water to a 5,000th of a grain of Corrosive Sublimate, we can "wash out" "the damned spot," and conclude that, because the discharge ceases, we have *cured* our patient? Perish the thought! This may occur in cases of simple urethritis, arising from exposure to cold or similar causes, but never from the gonorrhœal virus. The disease goes on all the same, in an endless variety of forms, many of which a majority of the profession have seemingly still to learn, and Dr. T. F. Allen, the editor of a work which ought to immortalize him, by his own admission, is one of the majority.

Yours fraternally,

Thos. Skinner, M. D.

